# Optimizing Stretchability and Electrical Stability in Bilayer-Structured Flexible Liquid Metal Composite Electrodes

**DOI:** 10.3390/mi15121467

**Published:** 2024-11-30

**Authors:** Min-Gi Kim, Kun-Woo Nam, Won-Jin Kim, Sung-Hoon Park

**Affiliations:** Department of Mechanical Engineering, Soongsil University, 369 Sangdo-ro, Dongjak-Gu, Seoul 06978, Republic of Korea; kimmingi4694@gmail.com (M.-G.K.); kwn1522@naver.com (K.-W.N.); dnjswls0214@gmail.com (W.-J.K.)

**Keywords:** liquid metal, stretchable electrode, bilayer, composite, piezoresistivity

## Abstract

Gallium-based liquid metals remain in a liquid state at room temperature and exhibit excellent electrical and thermal conductivities, low viscosity, and low toxicity, making them ideal for creating highly stretchable and conductive composites suitable for flexible electronic devices. Despite these benefits, conventional single-layer liquid metal composites face challenges, such as liquid metal leakage during deformation (e.g., stretching or bending) and limited elongation due to incomplete integration of the liquid metal within the elastomer matrix. To address these limitations, we introduced a bilayer structure into liquid metal composites, comprising a lower polydimethylsiloxane (PDMS) layer and an upper PDMS-liquid metal mixed layer. In the mixed layer, the liquid metal precipitates, forming a conductive network spanning both layers. This bilayer composite structure demonstrated significantly improved stretchability and elongation compared to pure PDMS or single-layer composites. Additionally, by adjusting the size and content of the liquid metal particles, we optimized the composite’s mechanical and electrical properties. Under optimal conditions, spherical liquid metal particles deform into elliptical shapes under tensile stress, increasing conductive pathways and reducing electrical resistance. The combined effects of the bilayer structure and particle shape deformation enhanced the composite’s stretchability and elongation, supporting its potential for flexible electronics applications.

## 1. Introduction

Stretchable electronic devices and wearable strain sensors are receiving increasing attention across various fields, including healthcare [1,2], robotics [3,4], and sports [5,6]. While traditional metal-based materials and electrodes exhibit exceptional electrical and mechanical properties, they face significant limitations in durability, elasticity, and reusability. Metal materials are prone to failure under repeated tensile deformation, and their deformation tends to accumulate over time, rendering them unsuitable for long-term applications [7,8,9]. To address these challenges, liquid metal-based composites [10,11,12,13] have emerged as promising candidates due to their superior conductivity, elasticity, and stability. Among these, gallium alloys, particularly Galinstan (GaInSn), are considered ideal materials [14] for fabricating flexible electrodes and sensors [15,16,17,18,19] due to their inherently high conductivity, remarkable flexibility, and compatibility with printing techniques. The ability to print liquid metal facilitates the fabrication of complex, customized structures, making it highly suitable for applications in stretchable and wearable electronics [20,21,22,23]. However, when exposed to air, an oxide layer rapidly forms on the surface of the liquid metal, impeding the conductive pathways. Restoring conductivity necessitates the removal of this oxide layer to reactivate its conductive properties [24,25]. Several approaches have been employed to achieve this restoration, including physical methods such as ultrasonic treatment and friction, chemical treatments to dissolve the oxide layer, and electrical stimulation to break the oxide layer [26,27,28]. Additionally, thermal treatment and laser sintering [29,30,31,32] can be utilized to decompose or remove oxide layers. Depending on the specific requirements, these techniques can be applied either independently or in combination to effectively restore the conductive properties of liquid metals.

**Figure 1:**
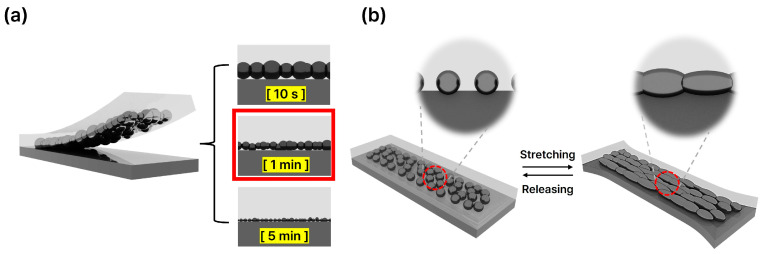
(**a**) Bilayer composite scheme. (**b**) Schematic illustration of the shape changes in liquid metal during tensile and compressive strain.

Figure 1a illustrates the overall structure of the bilayer liquid metal composite, along with the variation in liquid metal particle size as a function of sonication time. As sonication time increases, the size of the liquid metal particles decreases, directly influencing the composite’s electrical and mechanical properties. Figure 1b depicts the connections formed between liquid metal particles during tensile deformation, highlighting how the deformation of the liquid metal facilitates the formation of stable conductive pathways.

This study aims to optimize the performance of liquid metal composite electrodes by introducing a bilayer structure. The electrical performance and structural stability of the composites were evaluated by adjusting the size and content of the liquid metal particles. The optimized size and content of the liquid metal resulted in superior electrical conductivity, excellent mechanical properties, and enhanced long-term cycling stability. These findings provide valuable insights and practical guidelines for designing flexible electrodes using liquid metals.

## 2. Materials and Methods

### 2.1. Materials

Ethyl alcohol anhydrous (assay ≥ 99.9%) was purchased from SAMCHUN (Seoul, Republic of Korea). The primary material, Galinstan (GaInSn), was obtained from RND Korea (Gyeonggi, Republic of Korea). PDMS (SYLGARD 184 Silicone) was purchased from DOW CORNING (Midland, MI, USA) and used at an appropriate ratio.

### 2.2. Fabrication of Bilayer Liquid Metal Composites

Bilayer composites of Galinstan and PDMS were fabricated as follows: First, 5 g of Galinstan was added to 10 mL of ethyl alcohol and ultrasonicated at 70% intensity for 10 s, 1 min, and 5 min for each respective sample. After the Galinstan settled, the ethyl alcohol was removed, and the PDMS prepolymer (mixed with a curing agent at a 10:1 ratio) was added in proportions of 20 wt.%, 40 wt.%, and 80 wt.%. The mixture was stirred with a glass rod until the liquid metal was uniformly dispersed throughout the entire mixture. Subsequently, 1.65 g of PDMS (10:1) was applied to the bottom of a PTFE (Teflon) mold and cured in an oven at 80 °C for 20 min. Following this, 2 g of the previously prepared Galinstan–PDMS mixture was poured on top and cured at room temperature for 24 h.

### 2.3. Characterization and Test Conditions

To observe size differences in Galinstan based on sonication time, Galinstan was added to ethyl alcohol, sonicated, and then spin-coated prior to observation using a Gemini SEM 300 (ZEISS, Baden-Württemberg, Germany). The fractured surfaces of the bilayer composites were prepared by freezing the samples in liquid nitrogen, fracturing them, and observing them using SEM and an optical microscope (TUCSEN, Fuzhou, China).

All samples used for the measurements were fabricated in a PTFE mold with dimensions of 20 mm × 52 mm × 3 mm. A universal testing machine (UTM; Technology and Dorf, Gyeonggi-do, Republic of Korea) was employed to assess the mechanical properties, with tensile tests conducted at a speed of 50 mm/min under a load of 20 kgf.

For piezoresistivity measurements, a three-dimensional tensile machine (Namil, Incheon, Republic of Korea) was used to apply 100% and 200% strain for 10 cycles, with each cycle lasting 30 s. The changes in resistance during these cycles were analyzed using a Keithley DMM 7510 multimeter (Keithley, Cleveland, OH, USA).

A Fluke 114 multimeter (Fluke, Washington, DC, USA) was used to measure resistance changes before and after activation.

## 3. Results and Discussion

### 3.1. Morphology Analysis

**Figure 2:**
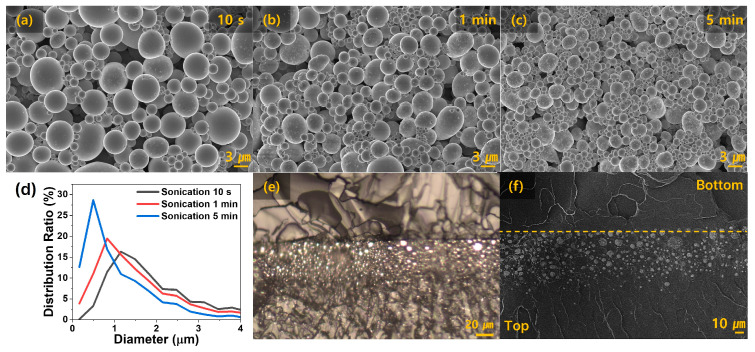
(**a**–**c**) SEM images of liquid metal particle size according to sonication time: 10 s, 1 min, 5 min. (**d**) Graph of particle size distribution according to sonication time. (**e**) Fracture surface observed using an optical microscope. (**f**) Dashed line indicates the boundary of the bilayer, with the fractured surface captured using SEM.

Figure 2a–c are SEM images illustrating the size of the liquid metal particles relative to the sonication time. As the sonication time increased, the applied energy caused the liquid metal to break down further into smaller particles, resulting in a gradual reduction in particle size. Figure 2d quantifies these differences, demonstrating that longer sonication times yield a greater number of smaller liquid metal particles in the overall distribution. Specifically, the distribution ratio for the largest particle diameter is 0.5 μm for 5 min, 0.8 μm for 1 min, and 1.2 μm for 10 s, respectively.

Figure 2e, captured using an optical microscope, shows the fracture surface of the bilayer composites, where the liquid metal precipitated between the PDMS layers. Figure 2f, obtained with SEM, provides a detailed view of the liquid metal’s precipitation toward the bottom of the sample, attributed to its higher density compared to PDMS. Additionally, larger particles settle more rapidly, concentrating at the bottom.

### 3.2. Mechanical and Electrical Properties

**Figure 3:**
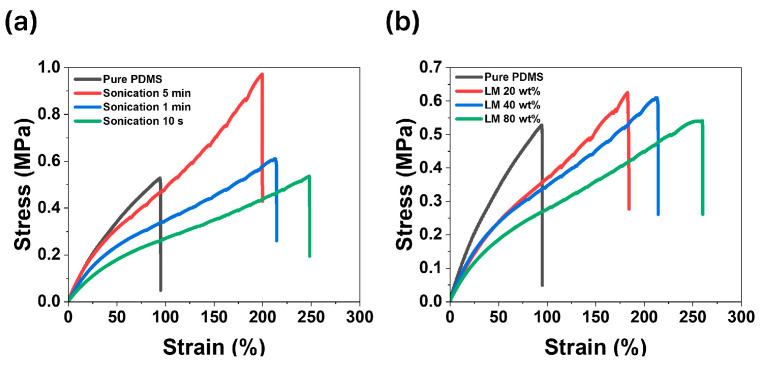
(**a**) Stress–strain curves of bilayer composites fabricated with varying sonication times. (**b**) Comparison of stress–strain curves of bilayer composites with different liquid metal content.

Figure 3a compares the mechanical properties of the bilayer composites fabricated with varying sonication times while maintaining the liquid metal (LM) content at 40 wt.%. Figure 3b compares the mechanical properties of composites with varying LM content while fixing the sonication time at 1 min. Thus, the sample labeled “Sonication 1 min” and the sample labeled “LM 40 wt.%” were fabricated under identical conditions. Figure 3a highlights how the mechanical properties vary with the liquid metal particle size, which is influenced by sonication time. The stress and strain values at fracture were 0.972 MPa and 199% for 5 min, 0.609 MPa and 214% for 1 min, and 0.536 MPa and 248% for 10 s, respectively. The corresponding Young’s moduli were 0.917, 0.680, and 0.491 MPa. As shown in Figure 2d, increasing the sonication time led to a higher proportion of smaller liquid metal particles, which increased the composite’s strength and Young’s modulus but decreased elongation at fracture. This indicates that liquid metal particle size significantly affects the mechanical properties of the composite, as smaller particles are more influenced by the solid oxide layer, causing the liquid metal to exhibit behavior closer to that of solid particles [33,34]. Figure 3b shows the stress–strain curves for composites with varying LM content. For the 20 wt.% and 80 wt.% LM samples, the stress and strain at fracture were 0.625 MPa and 183%, and 0.541 MPa and 260%, respectively. The corresponding Young’s moduli were 0.650 MPa and 0.532 MPa. As the LM content increased, elongation improved due to the decrease in Young’s modulus, which was directly influenced by the higher proportion of liquid metal in the composite [35].

**Figure 4:**
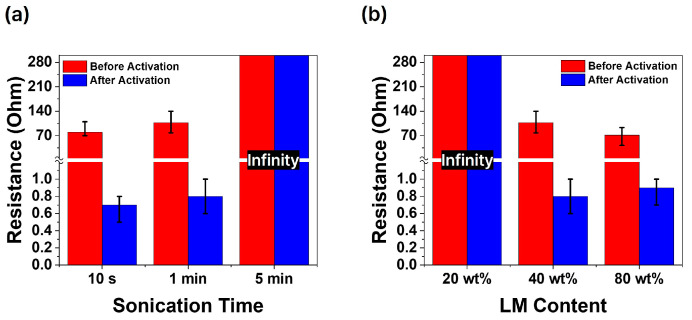
Comparison of resistance before and after activation (**a**) according to different sonication times and (**b**) based on different liquid metal content.

Figure 4a illustrates the resistance changes before and after activation for composites fabricated with different sonication times. As shown in Figure 2d, for the 10 s and 1 min samples, the proportion of smaller liquid metal particles was relatively low, allowing conductive pathways to form effectively before activation. After activation, the oxide layer was destroyed, leading to more continuous conductive pathways and a significant reduction in electrical resistance. In contrast, for the 5 min sample, the proportion of small particles (approximately 0.5 μm) was too high, hindering the formation of conductive pathways even after activation. Activation of the liquid metal–PDMS composite through compressive force caused small cracks in the PDMS, which then propagated rapidly under the intense stress exerted by the high-pressure liquid metal [36].

During this process, the liquid metal particles collide and break the oxide layer. For liquid metal particles with larger initial diameters, the penetration length into cracks is shorter. These larger particles are pushed into the cracks, inducing forces that prevent the composite from deforming further due to the cracks. Consequently, larger liquid metal particles enable greater current flow through the composite. In contrast, smaller particles exhibit less deformation under compressive force due to their high internal pressure and surface tension, which hinders their ability to penetrate the cracks in the PDMS. Additionally, the high ratio of oxide layer thickness to particle diameter makes breaking the oxide layer more challenging, thereby inhibiting the formation of conductive pathways [37,38]. Figure 4b presents a graph comparing the electrical resistance before and after activation for samples with varying liquid metal content, with the sonication time fixed at 1 min. Higher liquid metal content facilitates the formation of conductive pathways, and the activation process enhances the robustness of these pathways. However, in the case of the 20 wt.% composite, the absolute quantity of liquid metal was insufficient to form effective conductive pathways. Figure 4a indicates that the composite sonicated for 5 min is unsuitable because the smaller liquid metal particles make it difficult to establish conductive pathways. Similarly, Figure 4b shows that the 20 wt.% composite is inadequate due to the insufficient amount of liquid metal required to create reliable conductive pathways.

### 3.3. Piezoresistive Properties

**Figure 5:**
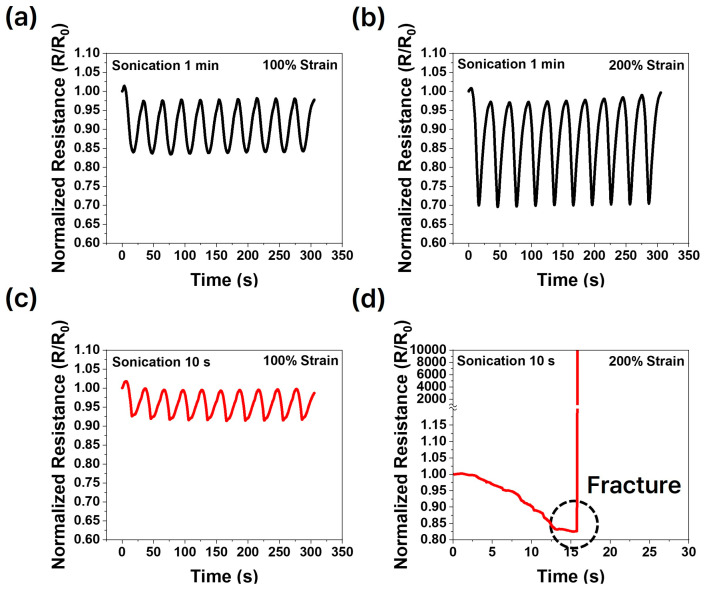
Normalized resistance change under tensile strain at different sonication times: (**a**) 1 min, 100% strain, (**b**) 1 min, 200% strain, (**c**) 10 s, 100% strain, (**d**) 10 s, 200% strain.

**Figure 6:**
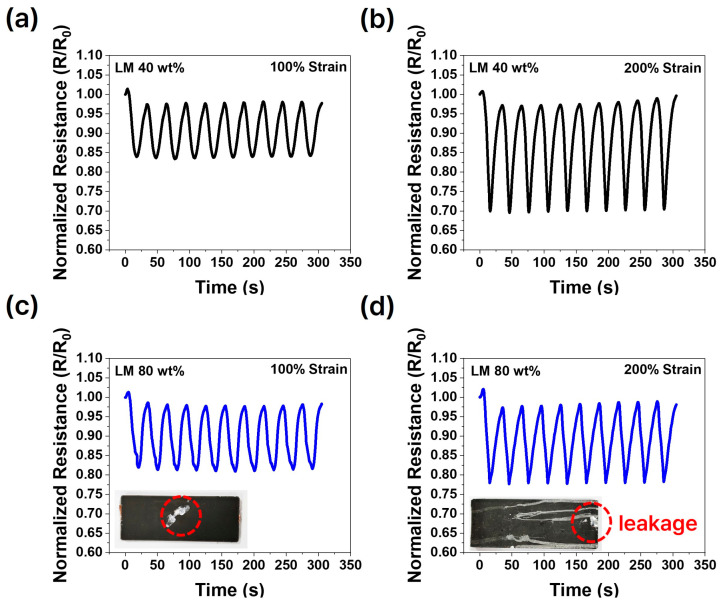
Normalized resistance change in relation to tensile strain with varying liquid metal contents: (**a**) 40 wt.%, 100% strain, (**b**) 40 wt.%, 200% strain, (**c**) 80 wt.%, 100% strain, (**d**) 80 wt.%, 200% strain.

Experiments were conducted to observe changes in resistance during the tensile process under varying liquid metal content and processing conditions. Each cycle lasted for a total of 30 s, comprising 15 s for stretching and 15 s for relaxation. The cycles were performed continuously without any waiting time between them. The samples depicted in Figure 5 and Figure 6 demonstrate a decrease in resistance during stretching and an increase in resistance during relaxation. In composites where the oxide layer has been broken and conductive pathways are established through activation, the liquid metal elongates into an elliptical shape under tensile strain. This deformation reduces the distance between particles and increases the contact area, thereby enhancing conductivity [39,40].

The expansion of conductive pathways during stretching resulted in a decrease in resistance. Conversely, during relaxation, the elliptical liquid metal particles returned to a spherical shape, increasing the distance between them and reducing the contact area, thereby leading to an increase in resistance. This behavior is illustrated in Figure 5 and Figure 6. Figure 5 highlights resistance changes based on processing differences, while Figure 6 focuses on resistance changes as a function of liquid metal content.

Figure 5a presents the resistance change when the composite, fabricated with 1 min of sonication, is subjected to 100% strain, whereas Figure 5b depicts the resistance change at 200% strain. At 200% strain, the larger deformation created more conductive pathways as the liquid metal underwent further deformation, shifting within the PDMS matrix and expanding the conductive pathways, resulting in a more pronounced resistance change.

Figure 5c,d show resistance change graphs for composites fabricated with 10 s of sonication and subjected to 100% and 200% strain, respectively. The larger liquid metal particles in the 1 min sonicated sample formed better connected conductive pathways during activation, leading to smaller resistance changes compared to the 10 s sample. As shown in Figure 5, both processes exhibited greater resistance changes at 200% strain compared to 100%. Specifically, Figure 5a indicates a 16% change, Figure 5b a 30% change, Figure 5c an 8% change, and Figure 5d a 17% change.

Although the normalized resistance change suggests a substantial increase based on the percentage values, the actual resistance change remained minimal, with variations in less than 1 Ω. While Figure 5c (100% strain) shows a trend similar to that in Figure 5a, the composite in Figure 5d (200% strain) fractured easily. The fracture occurred at approximately 15 s, coinciding with the point of maximum stretching. As demonstrated in Figure 3a, this failure can be attributed to the larger size of the liquid metal particles, which reduced Young’s modulus and mechanical strength, causing the sample to fail at the mounting points due to stress concentration during the stretching and relaxation tests.

Figure 6 illustrates the results of the stretching and relaxation tests as a function of the liquid metal (LM) content. Figure 6c,d reveal that samples with 80 wt.% LM experienced leakage, as indicated by the red circles, during both stretching and relaxation phases. This leakage underscores the challenge of maintaining structural integrity under mechanical strain when the liquid metal content is excessive.

As shown in Figure 1a, the composite features a bilayer structure. In conventional single-layer liquid metal composites [41], the high density of the liquid metal leads to sedimentation and leakage at the lower surface, even under minor deformation or tensile strain, making them highly prone to leakage. To address this, a bilayer structure was introduced to enhance the composite’s stability [42]. As demonstrated in Figure 6a,b, the bilayer structure effectively prevents leakage and enables stable cycling, even under mechanical stress. However, as seen in Figure 6c,d, an excessive amount of liquid metal overwhelms the structural benefits of the bilayer, leading to leakage during stretching and relaxation tests.

Figure 6 shows that, across all cases, resistance decreases during stretching and increases during relaxation. When leakage occurs, a significant and sudden shift in resistance is observed. At the moment of leakage, the conductive pathways are disrupted, resulting in a sharp increase in resistance. This disruption, however, is temporary, as the liquid metal tends to rearrange itself, allowing the resistance to gradually return to a state similar to its pre-leakage condition.

Although liquid metal may partially restore its conductive pathways after leakage, the recurring nature of this issue particularly in high-content samples renders these composites unsuitable for practical applications.

### 3.4. Hysteresis

Hysteresis is conventionally defined as the area enclosed by the loading–unloading curve in a stress–strain or resistance–strain plot, representing energy dissipation during cyclic deformation. However, in this study, the term “hysteresis” is used more broadly to describe the variation in electrical resistance between stretching and relaxation cycles, particularly as an indicator of the material’s ability to stabilize its electrical properties over repeated cycles.

Figure 4 illustrates that smaller liquid metal particles and lower content hinder the formation of conductive pathways, whereas Figure 5 shows that larger liquid metal particles lead to a reduced Young’s modulus and mechanical strength, making the material more susceptible to stress concentration and eventual fracture. Additionally, Figure 6 highlights that excessive liquid metal content induces leakage during the stretching and relaxation processes.

**Figure 7:**
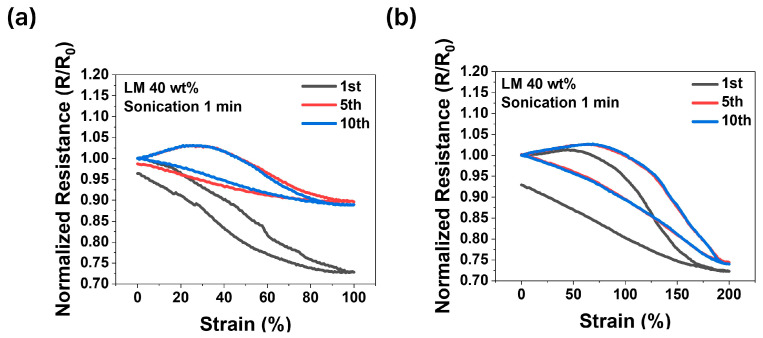
Hysteresis of 40 wt.% LM content samples fabricated under sonication for 1 min: (**a**) 100% strain, (**b**) 200% strain.

Hysteresis serves as a critical metric for assessing the ability of liquid metal composites to recover their original state after deformation and maintain the stability of their electrical properties. Accordingly, the hysteresis of the optimized sample (sonicated for 1 min with 40 wt.% liquid metal) was analyzed, as this sample demonstrates both structural stability and superior electrical conductivity. Figure 7a depicts the hysteresis behavior under 100% strain, while Figure 7b presents the results for 200% strain. Electrical resistance was monitored during the first, fifth, and tenth cycles of stretching and relaxation to evaluate changes in resistance over the cycles. In Figure 7a, hysteresis is apparent during the initial cycle but progressively decreases with subsequent cycles, becoming negligible by the tenth cycle. Conversely, Figure 7b shows that the deformation induced by 200% strain results in more pronounced hysteresis compared to 100% strain.

Despite the difference in hysteresis magnitude, both cases exhibit a consistent reduction in hysteresis with repeated cycles, eventually approaching near elimination. This phenomenon can be attributed to the initial misalignment of liquid metal within the composite, which gradually aligns along the stretching direction through repeated cycling, forming stable and robust conductive pathways. The minimal initial hysteresis observed under both 100% and 200% strain, combined with its rapid reduction over subsequent cycles, highlights the exceptional performance of this material as a high-quality electrode for stretchable electronics.

## 4. Conclusions

We developed electrodes with high electrical conductivity and stretchability by introducing a bilayer structure in liquid metal-based composites, effectively preventing liquid metal leakage and optimizing the size and content of liquid metal droplets. SEM analysis of the optimized composites revealed significant improvements in mechanical properties, including increased Young’s modulus and strength. The proper droplet size was critical for the formation of conductive pathways, as optimized droplets activated more pathways due to their ideal size. Smaller droplets, characterized by higher internal pressure and surface tension, resisted deformation and hindered pathway formation. Conversely, larger droplets exhibited a lower Young’s modulus, compromising structural stability during cyclic stretching. Resistance measurements before and after activation confirmed that conductive pathways formed effectively only when liquid metal particles exceeded a critical size. During tensile and relaxation cycles, the optimized composite demonstrated stable electrical resistance, while composites with excessive liquid metal content suffered from leakage. Hysteresis testing of the optimized 40 wt.% LM sample, sonicated for 1 min, confirmed low hysteresis and stable resistance under high tensile strain. In summary, bilayer composites optimized for liquid metal size and content exhibited stable electrical resistance, minimal hysteresis, and leakage-free performance during tensile cycles. This study identifies the optimal conditions for developing high-performance liquid metal electrodes in bilayer composites, providing a valuable framework for future advancements in stretchable electronics.

## Data Availability

The raw data supporting the conclusions of this article will be made available by the authors on request.
